# Innovative Use of Biodex Balance System to Improve Dynamic Stabilization and Function of Upper Quarter in Recreational Weightlifters: A Randomized Controlled Trial

**DOI:** 10.3390/medicina58111631

**Published:** 2022-11-11

**Authors:** Osama R. Abdelraouf, Amr A. Abdel-aziem, Shahesta A. Ghally, Lamis A. Osama, Reem S. Dawood, Amr M. Yehia, Emad M. Eed, Amira M. EI-Gendy, Rafik E. Radwan

**Affiliations:** 1 Physical Therapy Program, Batterjee Medical College, Jeddah 21442, Saudi Arabia; 2 Department of Physical Therapy, College of Applied Medical Sciences, Taif University, P.O. Box 11099, Taif 21944, Saudi Arabia; 3 Department of Musculoskeletal Disorders and Its Surgery, Faculty of Physical Therapy, October 6 University, Giza 12585, Egypt; 4 Department of Physical Therapy for Internal Medicine, Faculty of Physical Therapy, October 6 University, Giza 12585, Egypt; 5 Department of Clinical Laboratory Sciences, College of Applied Medical Sciences, Taif University, P.O. Box 11099, Taif 21944, Saudi Arabia; 6 Department of Basic Science for Physical Therapy, Faculty of Physical Therapy, Cairo University, Giza 12613, Egypt; 7 Department of Biomechanics, Faculty of Physical Therapy, Cairo University, Giza 12611, Egypt

**Keywords:** Biodex, functional performance test, recreational weightlifters, upper quarter

## Abstract

*Background and Objectives*: Following an injury, upper-body strength and proprioception training is typically suggested. To our understanding, no prior research has looked into the impact of balance training on upper-body strength and stability. So, this study investigated the effects of Biodex balance training on enhancing the dynamic stability, strength, and function of the upper quarter (UQ) in recreational weightlifters. *Materials and Methods*: Fifty male weightlifters were randomly assigned into two groups. The experimental group received an upper-extremity Biodex balance training program three times/week for eight weeks, while the control group underwent a regular weightlifting training routine. Pre- and post-test scores of the upper-quarter dynamic stability, strength, and function were measured for both groups using the shoulder active repositioning accuracy test, two-minute push-up test, and the upper-quarter Y-balance test (UQ-YBT) and one-arm hop test, respectively. *Results*: Post-test values were significantly greater for the normalized UQ-YBT test than pre-test values in both groups (*p* < 0.05). Post-test values of the experimental group were significantly greater than the control group (*p* < 0.05). Regarding the shoulder active repositioning accuracy test and the time of the one-arm hop test, post-test values were significantly lower than pre-test values for both groups (*p* < 0.05), and post-test values of the experimental group were significantly lower than those of the control group (*p* < 0.05). The post-test value of the two-minute push-up test of each group was significantly higher than the pre-test value (*p* < 0.05), without any significant difference between both groups (*p* > 0.05). *Conclusions*: Adding upper-body Biodex balance training to a regular weightlifting training routine was effective in enhancing the upper quarter′s dynamic stability and function.

## 1. Introduction

Recreational weightlifting has become an increasingly popular physical activity, typically performed to achieve muscular hypertrophy, increase strength, and gain an attractive body physique [1,2]. At least 45 million people across the United States actively participate in weightlifting programs [3]. Repeated lifting of enormous weights during this type of training can cause high moments, compressive loads and shearing forces on the spine and joints with increased intrathoracic and intraabdominal pressures [4,5].

The shoulder complex is a common region for exercise-related injuries, comprising 36% of all incidents [6]. Core muscles endurance should be emphasized to reduce the injury risk [7]. Associated factors with these injuries are technical errors, fatigue with a consequent decline in proprioception, overloading, and dropping weights. Although each injury is different in terms of anatomic position and mechanism, they are all preventable if risk factors are properly managed [8].

Closed kinetic chain (CKC) assessment tools to identify deficits in the upper extremity are essential for the description of effective training programs [9]. Unfortunately, there are several limitations of the reported upper-extremity CKC tests that were reported in the literature. This could explain why no widely recognized clinical evaluation method for upper-body closed-chain performance exists [10,11]. The CKC upper-extremity stability test’s chief drawback is its inability to differentiate the performance of a single limb, as it requires both limbs to function simultaneously [12]. Other tests involving side bridging and planks are purely static tests [13,14].

Lately, the upper-quarter Y-balance test (UQ-YBT) has been established as a CKC evaluation for the upper-extremity (UE) and the entire upper-quarter (UQ) mobility and stability. Testing the body regions as quadrants can be an effective and comprehensive approach to explore severe deficits in performance, strength, or mobility [15]. Independent upper-limb evaluations are possible with the UQ-YBT, allowing comparisons between injured and noninjured limbs. The UQ-YBT was created because no other test had previously been noted that evaluated the joint stability and mobility of the whole UQ as well as the trunk at the limit of stability [14,16].

Moreover, the one-arm hop test is used for research purposes to measure the upper quarter′s functional performance. This test requires a great deal of eccentric and concentric muscle control while the upper extremity′s distal portion is under extreme axial load [17]. An extended testing battery might also include the two-minute 90° push-up test that is commonly used as a standard measurement for upper-body muscular strength [18]. It has lately also been proposed to exhaustion to objectively evaluate the upper-body endurance in the physical fitness field [19].

Balance and proprioception training are types of neuromuscular interventions that could be used by recreational athletes to improve their posture, strength, coordination, and joint stability during weightlifting practices. The alteration in the relation between the center of gravity and base of support or postural sway determines the training′s difficulty level [20]. Many devices are used for stability training of the upper extremity, including air-pressurized balls, both-sides-up (BOSU) balls, wobble boards, and foam platforms with the purpose to provide an unstable surface while performing different challenging tasks [21]. Unluckily, none of these devices provide visual feedback during training or a numeric score that represents the level of performance at the end of every session.

The Biodex balance system (BBS) has a wide range of clinical applications for balance training and evaluation. It has been recognized as an important tool for balance and postural stability training in various clinical settings [22]. Previous studies found the BBS effective in improving postural balance in patients with multiple sclerosis, Parkinson disease, stroke, diabetic neuropathy and the elderly population who are at risk of falls [23,24,25,26,27]. Although many studies have been conducted, no previous research has investigated the effectiveness of UQ proprioception and dynamic stability training using the Biodex balance system. This study was performed to fill a gap in the literature by investigating the effect of upper-extremity Biodex balance training on enhancing the dynamic stability, strength, and function of the UQ in recreational weightlifters.

## 2. Materials and Methods

### 2.1. Participants

**Figure 1:**
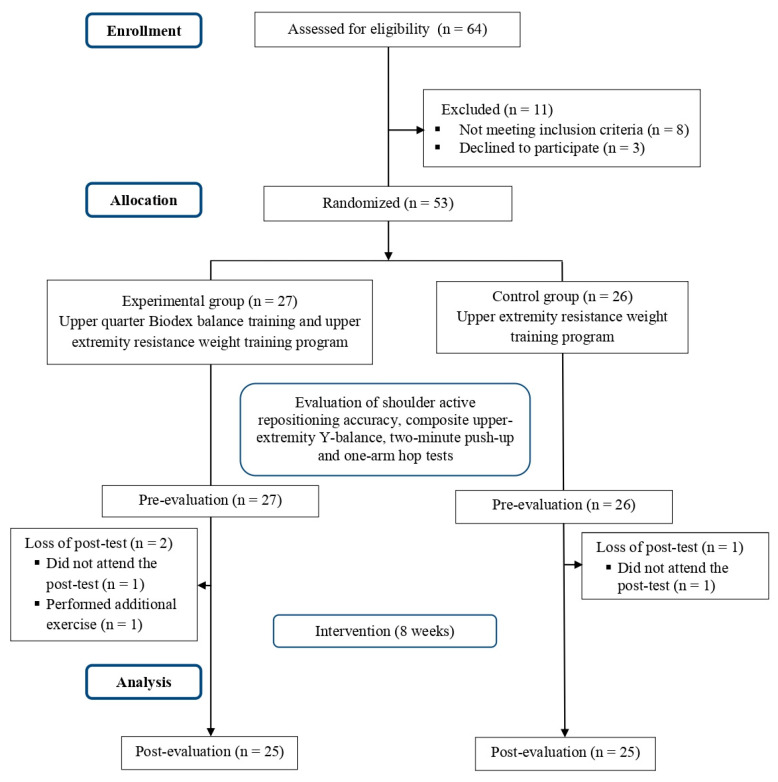
Flowchart of the study processes.

This single-blinded, randomized controlled study was conducted between January and April of 2021. Sixty-four recreational weightlifters, aged between 19 and 25 years, recruited from a local gymnasium, were initially validated for eligibility; 11 out of them did not undergo the inclusion criteria. The remaining 53 weightlifters were randomized into two groups: the experiment group (n = 27), and the control group (n = 26). The participants′ mean age, height, body mass index (BMI), and upper-extremity length were 22.52 years, 137.74 cm, 78.29 kg, 25.94 kg/m^2^ and 71.74 cm, respectively. Post experimental measurements were collected from 50 participants only, as three weightlifters were excluded for different reasons, as shown in Figure 1.

All participants were involved in upper-body resistance weight training an average of three days per week at 75–85% of their one repetition maximum (1RM) for 3–4 sets of 7–9 repetitions with a minimum duration of two years of practice [28]. They were excluded from the study if they used hormonal supplements, competed in powerlifting competitions or professional bodybuilding, had shoulder pain, musculoskeletal injuries, or a history of surgery, or missed more than three weeks of gym workouts in the previous six months.

Participants were assigned using a computer random-generated number list into group A (experimental), who received UQ Biodex balance training three times/week for eight weeks. Group B (control) only continued their weightlifting routine. Individuals in both groups were permitted to continue their weightlifting routine during the period of the study. The random distribution was conducted by an independent researcher who was not aware of the procedures of the trial and has no contact with the participants. G*Power 3.1.9.4 software (Universität Düsseldorf, Düsseldorf, Germany) was used to calculate the sample size. The effect size (ES) was determined using the test′s value and power. The total sample size was estimated to be 36 participants using ES (0.25), α(0.05), and power of the test (0.95).

Each participant signed a written informed consent form. The Faculty of Physical Therapy of Cairo University Institutional Review Board authorized the study protocol (Approval No. PT.REC.012.002982), and it was registered with ClinicalTrials.gov PRS (NCT04670861). The research was performed in line with the Declaration of Helsinki principles.

### 2.2. Testing Procedures

To prevent the effect of fatigue on the respondents′ physical performance, data from the testing protocols in the pre- and post-interventional phases were collected over two days. On the first day, descriptive data such as age, weight, and upper-extremity dominance were measured for each subject (known as the hand noted to be favored for ball throwing). The UQ-YBT and the shoulder active repositioning accuracy test were then determined utilizing the isokinetic dynamometer. On the second day, data from the two-minute 90° push-up test and the one-arm hop test were gathered. For all participants, the order of the days and the testing protocols were randomized. For all tests, participants completed two familiarization trials first before the actual measurements were recorded.

### 2.3. Upper-Quarter Y-Balance Test

**Figure 2:**
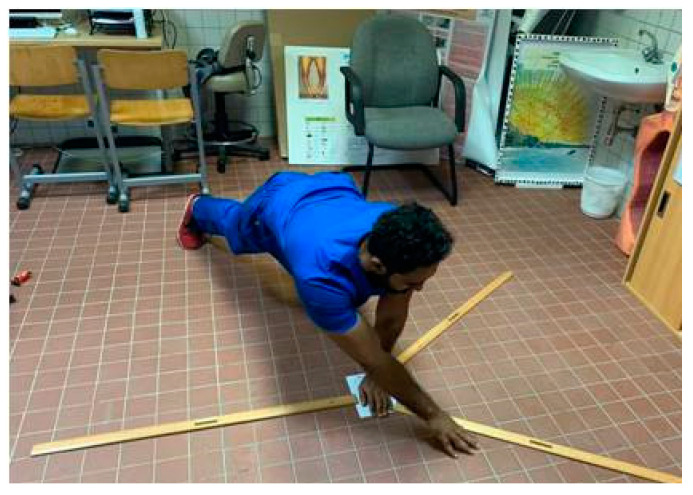
Upper-quarter Y-balance test.

The UQ-YBT consists of a platform with three wooden rulers connected to it in the medial, superolateral, and inferolateral reach directions. Each of the posterior rulers is at an angle of 135 degrees to the anterior ruler. A 90-degree angle between them separates the posterior rulers. Each ruler has the marks of 0.5-cm increments for measurement. Three familiarization trials for each side were provided before the actual testing, in which participants were instructed to put one hand on the platform and reach with the other hand as far as they could in each testing direction. The maximum reach distances in the medial, superolateral, and inferolateral directions were observed and recorded by a research assistant, who was blinded to the aim of the study (the average of three trials), in centimeters (cm) (Figure 2). Finally, the test results were normalized to arm′s length of the participants by dividing the reach distance sum in the three directions by three times the upper-extremity length, which was recorded from the acromion process to the tip middle finger with a straight elbow and wrist, then multiplying the result by 100. Data normalization is important to avoid the arm length′s influence on test performance [29]. The test–retest reliability of the UQ-YBT varied from 0.80 to 0.99 while the intraclass correlation coefficient for interrater reliability was 1.00 [16].

### 2.4. Shoulder Active Repositioning Accuracy Using Isokinetic Dynamometer

Based on the conclusion of a 2017 systematic review, the evaluation of shoulder proprioception using an isokinetic dynamometer is valid, and most reliable when using a protocol for internal rotation at 90° of shoulder abduction [30]. A Biodex isokinetic dynamometer (Biodex Medical Inc., Shirley, NY, USA) was used for measuring the shoulder repositioning accuracy. Each participant sat in a comfortable sitting position on the Biodex chair, with the trunk upright and the hips and knees at about 85° flexion. A firm back supported the trunk up to the scapular level and stabilized it with a pelvic strap and a pair of anterior straps stretched diagonally from just above the shoulder level to the opposite pelvic side. A single strap was placed horizontally across the tested side′s thigh. The arm was abducted 90 degrees in the scapular plane and supported. Each participant underwent two familiarization trials prior to data collection, the first with the eyes open as the tested extremity was passively moved to the target angle of 75 degrees of shoulder internal rotation, and then it was held for 10 s as a teaching process for the participant so that he or she could memorize the position. The apparatus then allowed the limb to return to its original position. After a 30-second rest, the participant was allowed to actively return to the target angle of 75 degrees with the eyes closed. When the participant felt he had reached the target angle actively, he asked the examiner to stop the apparatus. Three trials were recorded for each participant. The angular difference between the target angle position and participants’ perceived end-range position was recorded in degrees as the deficit in repositioning accuracy [31]. Measurements were taken for the nondominant shoulder, because it is more accurate than the dominant shoulder when moving into internal rotation [32].

### 2.5. Two-Minute (2 Min) 90° Push-Up Test

All participants were given a familiarization session through which the test procedures and the scoring system were fully explained. Several uncounted trials were allowed as a warm-up before the actual testing took place. With hands slightly wider than shoulder width and fingers pointing forward, the exercise was performed on a flat, stable surface. The push-up is started by bending the elbows and minimizing the entire body as a single unit until the upper arms are at least parallel to the ground (90° push-up), and then the push-up is finished by elevating the entire body until the arms are fully extended. The goal of this test is to complete as many repetitions as possible in two minutes [18]. Throughout the movement, subjects were instructed to preserve a neutral spine and a feet-together position. The inter-rater reliability of the test was reported to reach up to 0.97 and the intra-rater reliability up to 0.99 [33].

### 2.6. One-Arm Hop Test

**Figure 3:**
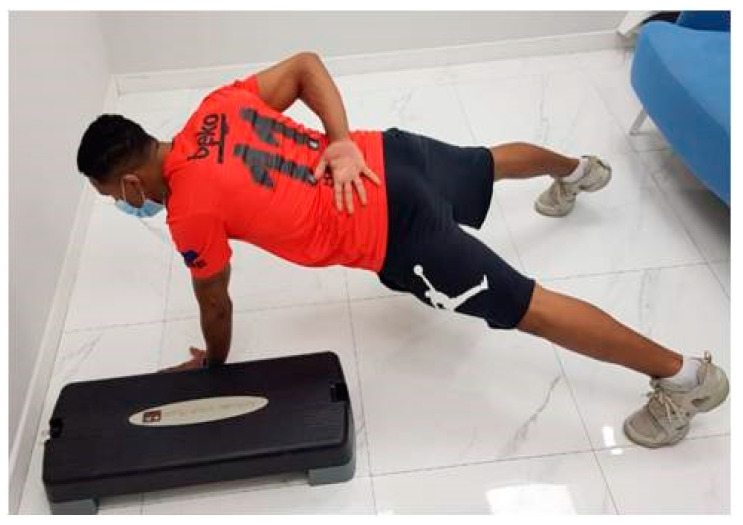
Starting position of the one-arm hop test.

The test procedures were explained in full detail to all participants. The subjects were given two sets of ten standard two-hand push-ups to warm up, with a two-minute rest period in between. The one-arm hop test was then conducted with the nondominant and dominant upper extremities in familiarization trials. The subject began by demonstrating the one-arm hop test on video. The actual testing started after a 10 min rest. Each subject performed a one-arm push-up with his back flat, feet shoulder-width apart, and weight-bearing upper extremity perpendicular to the floor. The non-weight-bearing hand was placed on the posterior aspect of the subject′s low back. A step with 10 cm height was placed just lateral to the subject′s weight-bearing hand. The subject used his arm to hop onto the step and back into the floor (Figure 3). The time needed to repeat this movement five times as quickly as possible was recorded using a stopwatch. The same maneuver was conducted with the contralateral upper extremity after a one-minute rest. The order in which the dominant and nondominant upper extremities were practiced was randomized. The test–retest reliability was reported to be as high as 0.81 and 0.78 for male football players and collegiate wrestlers, respectively [17].

### 2.7. Training Procedure

**Figure 4:**
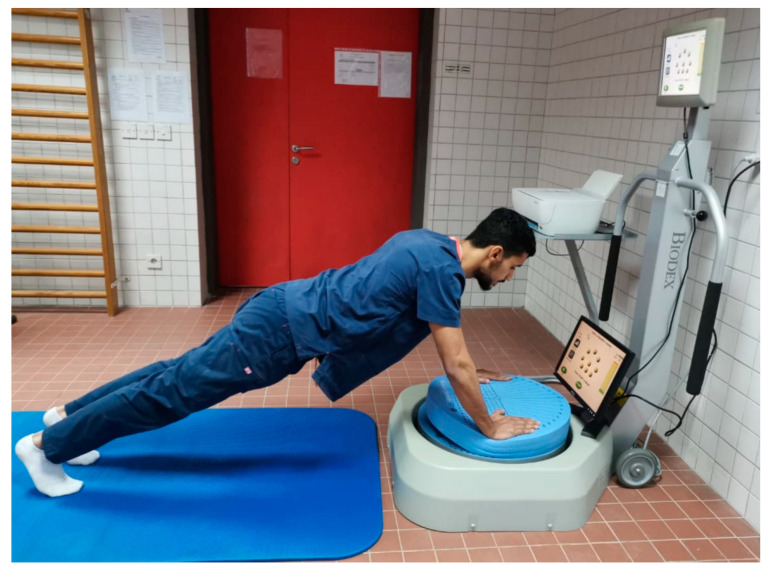
Biodex Balance System (BBS), training procedures.

The participants in Group A were scheduled to attend 24 training sessions over the course of eight weeks (three sessions per week). Training began with each participant′s present level of balance, at which he reveals an exertion level of ≥5 on a modified Borg scale [34,35], and progressed towards the more complicated ones. The Biodex display screen was linked to a computer screen that was positioned at a level that allowed participants to receive visual feedback throughout training. The BBS is a percent score out of one hundred; the higher the score, the better the dynamic stability. A composite score of more than 95 percent at the current level was used to determine the transition from one level to the more difficult one. Each training session lasted 20 min and comprises three minutes of training and one minute of rest (Figure 4). However, on improvement progression, using a longer training time with the same rest intervals was implemented [36].

### 2.8. Statistical Analysis 

A Statistical Package for Social Sciences (SPSS) for Windows version 20.0. (SPSS, Inc., Chicago, Illinois) was utilized to analyze the obtained data. Prior to the final statistical analysis, the test of homogeneity (Levene′s test) showed that there were no significant differences between the variances of the experimental and control groups of the tested dependent variables (*p* = 0.593), and the Shapiro–Wilk test demonstrated that the data were normally distributed (*p* = 0.372). The significant differences in the mean values of height, age, weight, or BMI between both groups were measured by utilizing the independent *t*-test. The differences between the shoulder active repositioning accuracy test, composite right/left upper-extremity Y-balance, two-minute push-up and right/left one-arm hop tests of both groups were tested by using the multivariate ANOVA (MANOVA) to determine main effect of time and group (2 × 2) for each parameter. Then, Tukey′s test had been performed in case of significant results. The source of differences determined by utilizing the least significant difference test with an alpha level of 0.05 was regarded to be statistically significant for all tests.

## 3. Results

Table 1 indicated that there were no significant differences between both groups in height (*p* = 0.234), age (*p* = 0.373), BMI (*p* = 0.635), and weight (*p* = 0.379). Descriptive statistics of the balance composite score, and medial, superolateral, inferolateral directions of the UQ-YBT scores before and after the training program, are demonstrated in Table 2.

Regarding the measured outcomes, there was a statistically significant difference in the between-subjects effect (groups; F = 3.37, *p* = 0.002), and a significant difference in the within-subjects effect (time; F = 8.17, *p* = 0.001), and the interaction effect (time*group; F = 41.12, *p* = 0.001). Subsequently, multiple pairwise comparison tests were performed to characterize the significance source as per group interactions (intervention versus control) and time (pre versus post) factors.

Concerning the shoulder active repositioning accuracy, there were no significant differences between the pre-test values of the experimental and control groups (*p* = 0.333). The pre-test values of both groups were significantly lower than their post-test values (*p* = 0.001). The post-test value of the shoulder active repositioning accuracy of the experimental group was significantly higher than the control group (*p* = 0.019).

For the right and left UQ-YBT normalized scores, there were no significant differences between the pre-test values of the experimental and control groups (*p* = 0.302, 0.201, respectively). The pre-test values of both groups were significantly lower than their post-test values (*p* = 0.001). The post-test values of the right and left UQ-YBT normalized scores of the experimental group were significantly higher than control group (*p* = 0.025, 0.003,respectively).

Regarding the two-minute push-up test, there were no significant differences between the pre-test of the experimental and control groups (*p* = 0.245). The post-test values of each group were significantly greater than the pre-test values (*p* = 0.001), with no statistical difference between the post-test values of both groups (*p* = 0.077).

For the right and left one-arm hop test, there were no significant differences between the pre-test values of the control and experimental groups (*p* = 0.632, 0.818, respectively). Each group′s post-test values were significantly less than during pre-test (*p*= 0.001). The post-value of the right and left one-arm hop test of the experimental group was significantly lower than the control group (*p* = 0.002, 0.007), as illustrated in Table 2.

## 4. Discussion

The BBS has been used to provide a valuable balance analysis in a wide variety of musculoskeletal, vestibular, and neurogenic conditions. In the field of sports medicine, the system is ideal for pre-season balance screening to make injury predictions or to select the best injury prevention strategies, and as an efficient tool in return-to-sport decision-making [21]. Recently, BBS gained attention in weight-bearing balance training, closed chain for lower-extremity patients. Utilizing this unique device, clinicians can enhance kinesthetic abilities to compensate for the impaired proprioception following injuries or diseases [34,37].

To our understanding, this is the first study to assess the impacts of BBS training on the strength, dynamic stabilization, and functional performance of the upper quarter. The novel training approach used in this study increased the reaching distance of UQ-YBT in all directions and improved the shoulder repositioning accuracy in young recreational weightlifters as compared to controls. However, there was no statistical difference in upper-extremity strength as measured by the two-minute push-up test between both groups. 

The results of the current study could be attributed to several factors. The main reason for improved dynamic stabilization and the UQ′s functional performance in the experimental group was attributed to training on an unstable service. In young healthy adults, using unstable surfaces (STU) for a seven-week strength training resulted in enhanced strength, stability, and physical performance [38]. Furthermore, the Canadian Society for Exercise Physiology position stand [39] suggested that instability resistance training can play a significant role within a periodized training schedule for athletes as well as for nonathletic individuals to accomplish musculoskeletal health benefits. The soft nature of foam cushions contributes to postural disequilibrium, because the surface distorts in response to the reaction force related to alterations in the center of pressure [40]. Further, a greater activation of the lumbo–pelvic–hip muscles was measured during the performance of squat exercises with a balance disc under each foot as compared with the performance of the same movement on a stable surface [41].

From another perspective, the systematic review and meta-analysis conducted by Behm et al. [40] concluded that the specific comparison of STU with strength training on a stable surface (STS) in adolescents and young adults culminated with contradictory findings, and thus the unstable surfaces′ use for training was not recommended in this specific population. The difference in methodological approaches used in the studies included in the previously mentioned systematic review may account for these contradictory findings. However, there was neither a consistent training protocol to be utilized nor a standardized assessment tool.

For example, Sparkes and Behm [42] showed no significant training-specific differences on electromyographic activity of the triceps brachii and pectoralis major, chest press isometric force, balance, one-legged throwing distance, and drop jump (DJ) heights after eight weeks of STU and STS. The small sample size, 18 participants, that was allocated equally into two groups, is a major limitation that might threaten the external validity of their study. Furthermore, none of these participants had enrolled in a resistance training program in the previous two years. The difference in neuromuscular and functional adaptations between trained and untrained individuals might add another reason for the contradiction with the results of our present study. Similarly, the study of Maté-Muñoz et al. [43] showed that conventional and an instability resistance circuit training program had similar impacts on upper- and lower-limb strength, power, movement velocity, and jumping ability. The subjects in this study were physically active people who were unfamiliar with or had little experience with resistance training. The authors stated that their findings cannot be applied to high-performance athletes or subjects with prior resistance-training experience.

The lack of superiority of STU reported in the literature brings to attention the importance of the training intensity–response relationship. The degree of instability should be high enough to elicit a significant difference in the pre–post measured variables [40]. Unfortunately, there are no specific studies contrasting the extent of center of pressure excursions with various unstable surfaces (e.g., a BOSU ball, Resist-A-Ball, balance pad, Dyna Disc, Swiss ball, T-Bow). None of the previous studies have used the Biodex balance system as an objective tool to provide instability training. In our present study, the challenge of training instability was intensified by adding a thick foam pad on the top of the tilting balance platform to meet the high physical capabilities of the selected population.

The inability of the BBS training to improve the UQ strength in the experimental group can be understood by the fact that the STU induces a 30% deficit in strength as compared with the STS. This deficit happens due to lower external loads or torques and could be viewed as a protective mechanism to reduce the incidence of training-induced injuries [21]. Because young people have higher levels of circulating androgens, high training loads seem to be more appropriate for inducing a significant elevation in muscle strength in this age group. The reported improvement in strength in both the experimental and the control groups could be attributed to the resistance training where high weights are lifted rather than the BBS training.

An electromyography study [12] has looked at how the use of stable and unstable surfaces affects the stimulation pattern of the scapular and upper-limb muscles throughout push-ups plus beyond. A decreased electromyography activity of the anterior and posterior deltoid was observed while executing the exercise on an unstable surface, while those of the scapular stabilizing muscles are increased. This specific activation pattern might explain why dynamic stabilization, but not strength, has improved after the implementation of the BBS training program in the current study.

Training specificity is another factor in the improved dynamic stabilization and function of the UQ in the experimental group. As per the principle of training specificity, training should mimic the needs of the job or exercise as precisely as possible. Willardson [44] mentioned that practicing a skill on the same surface on which it is conducted is the best way to improve proprioception, balance, and spinal stability for any given sport. Although weight lifting is performed on stable surfaces, it provides a moderate-to-high degree of instability depending on the weight of the carried loads. External disruptive torques result from the movement of bars and dumbbells on the shoulders (such as squats), above the body (such as cleans, shoulder presses, snatches), or in front of the body (i.e., bicep curls), attempting to displace the center of gravity outside the foundation of the base of support, challenging the entire system to keep equilibrium [21].

Finally, the presence of visual feedback and performance scoring after each trial was a unique feature of using BBS training to improve stabilization and function of the upper body. Unfortunately, most of the other training tools lack this important motivational element of training [45]. Xu et al. [46] noted that, according to the findings, both the visual and proprioceptive systems influence function and balance, with vision playing a more important role. As a consequence, researchers came to the conclusion that combining visual feedback balance training with traditional rehabilitation could produce better results than traditional rehabilitation alone [47]. The findings of this study should encourage physical therapists and fitness coaches to include BBS training as an integral part of injury prevention and performance enhancement programs. The system is a valuable training tool for improving kinesthetic abilities, which may provide some compensation for impaired or delayed proprioceptive reflex mechanisms. Furthermore, BBS provides objective printed reports that document the athlete′s needs, progress, and outcomes [48,49]. This study has three main limitations. First, it was conducted on male recreational weightlifters only, as female candidates did not meet the inclusion criteria. Further research is recommended to explore the cross-gender effects of BBS training on the strength, power, stabilization, and function of the upper quarter. Second, the sample studied was comprised of healthy recreational weightlifters, and the results may not translate to pathologic populations. Therefore, the study should be duplicated in different populations with various upper-extremity musculoskeletal injuries and postoperative shoulder patients in later phases of rehabilitation. Third, the fact that the interventional group had a longer training activity duration could in part explain the difference between the groups. A similar training duration could be useful to show the plus value of BBS intervention. The last limitation is the inability of the two-minute (2 min) 90° push-up test to differentiate performance for a single limb. In consequence, comparison between both limbs was not possible. Moreover, an electromyographic (EMG) study is also needed to determine the muscle activation pattern during upper-quarter Biodex training.

## 5. Conclusions

The novel approach of BBS training for the upper body is effective in improving the dynamic stabilization and functional performance of the UQ in young recreational weight lifters. However, this was not the case in terms of UQ strength. Future studies are recommended to explore the muscle activation pattern during BBS training.

## Figures and Tables

**Table 1:** Demographic data of the participants.

	Experimental Group Mean ± SD	Control Group Mean ± SD	*p* Value
Age, year	22.24 ± 2.15	22.80 ± 2.25	0.373
Height, cm	174.56 ± 4.54	172.92 ± 4.37	0.234
Weight, kg	79.32 ± 8.10	77.36 ± 7.49	0.379
BMI, kg/m^2^	26.06 ± 2.00	25.82 ± 1.59	0.635
Upper-extremity length, cm	72.12 ± 4.59	71.36 ± 3.56	0.516

SD: Standard deviation, BMI: body mass index.

**Table 2:** The pre and post values of the Biodex balance and composite upper-quarter Y-balance test scores of both groups.

		Experimental Group Mean ± SD	Control Group Mean ± SD	*p* Value
Shoulder active repositioning accuracy test (degree)	pre	5.23 ± 2.52	5.98 ± 2.83	0.333
	post	3.21 ± 1.97	4.06 ± 2.04	0.019 *
	*p* value	0.001 *	0.001 *	
Composite right upper-extremity Y-balance test score (cm)	pre	82.47 ± 10.68	79.24 ± 9.47	0.302
	post	91.57 ± 10.20	84.92 ± 10.20	0.025 *
	*p* value	0.001 *	0.001 *	
Composite left upper-extremity Y-balance test score (cm)	pre	80.46 ± 9.97	77.52 ± 7.57	0.201
	post	90.90 ± 10.47	83.27 ± 6.57	0.003 *
	*p* value	0.001 *	0.001 *	
Two-minute push-up test (no. of repetitions)	pre	53.96 ± 4.49	55.52 ± 4.87	0.245
	post	67.24 ± 5.75	70.16 ± 5.68	0.077
	*p* value	0.001 *	0.001 *	
Right one-arm hop test (seconds)	pre	6.78 ± 1.25	6.60 ± 1.38	0.632
	post	4.26 ± 1.18	5.36 ± 1.22	0.002
	*p* value	0.001 *	0.001 *	
Left one-arm hop test (seconds)	pre	6.18 ± 1.07	6.10 ± 1.35	0.818
	post	4.08 ± 1.07	5.02 ± 1.26	0.007
	*p* value	0.001 *	0.001 *	

SD: Standard deviation, * means significant difference (*p* < 0.05).

## Data Availability

Data available on request.
